# Regulators of G-Protein Signaling (RGS) in Sporadic and Colitis-Associated Colorectal Cancer

**DOI:** 10.3390/ijms25010577

**Published:** 2024-01-01

**Authors:** Mikolaj Swierczynski, Zuzanna Kasprzak, Adam Makaro, Maciej Salaga

**Affiliations:** Department of Biochemistry, Faculty of Medicine, Medical University of Lodz, Mazowiecka 5, 92-215 Lodz, Poland; mikolaj.swierczynski@stud.umed.lodz.pl (M.S.); zuzanna.kasprzak@stud.umed.lodz.pl (Z.K.); adam.makaro@stud.umed.lodz.pl (A.M.)

**Keywords:** colitis-associated colorectal cancer, inflammation, inflammatory bowel disease, G-protein coupled receptors, regulators of G-protein signaling, AXIN, cannabinoid receptors, opioid receptors, serotonin receptors

## Abstract

Colorectal cancer (CRC) is one of the most common neoplasms worldwide. Among the risk factors of CRC, inflammatory bowel disease (IBD) is one of the most important ones leading to the development of colitis-associated CRC (CAC). G-protein coupled receptors (GPCR) are transmembrane receptors that orchestrate a multitude of signaling cascades in response to external stimuli. Because of their functionality, they are promising targets in research on new strategies for CRC diagnostics and treatment. Recently, regulators of G-proteins (RGS) have been attracting attention in the field of oncology. Typically, they serve as negative regulators of GPCR responses to both physiological stimuli and medications. RGS activity can lead to both beneficial and harmful effects depending on the nature of the stimulus. However, the atypical RGS—AXIN uses its RGS domain to antagonize key signaling pathways in CRC development through the stabilization of the β-catenin destruction complex. Since AXIN does not limit the efficiency of medications, it seems to be an even more promising pharmacological target in CRC treatment. In this review, we discuss the current state of knowledge on RGS significance in sporadic CRC and CAC with particular emphasis on the regulation of GPCR involved in IBD-related inflammation comprising opioid, cannabinoid and serotonin receptors.

## 1. Introduction

Colorectal cancer (CRC) is one of the most common neoplasms in both male and female populations worldwide. According to the American Cancer Society [1], the lifetime risk of developing CRC is estimated at 4.3% for men and 4.0% for women, being responsible for 10% of new cases and 9.4% of total cancer deaths in 2020 [2]. Unfortunately, the morbidity of this disease is expected to continuously increase in the future. The significance of CRC morbidity in the general population is emphasized not only by epidemiological data but also by the clinical image of this disease. It includes symptoms significantly decreasing patients’ quality of life that can be classified based on origin: (1) primary tumor growth responsible for local manifestations, such as intestinal obstruction, bleeding or pain, (2) metastases resulting in organ-specific malfunctions, (3) general malnutrition caused by metabolic burden and gastrointestinal (GI) tract impairment. The etiology of CRC is multifactorial and includes both genetic predisposition (e.g., Lynch syndrome, familial adenomatous polyposis) and environmental factors, including aging, high-caloric diet, smoking and insufficient physical activity [3]. However, a specific subtype of CRC, the so-called colitis-associated colorectal cancer (CAC), can also originate from chronic inflammation in the course of inflammatory bowel diseases (IBD), including Crohn’s disease (CD) and especially ulcerative colitis (UC) [4]. It was estimated that in the group of IBD patients, the lifetime risk of CAC development is increased up to 20% [5]. Differences in the etiology of CAC and sporadic CRC lead to further alterations in basic aspects of carcinogenesis, including mutation patterns and dynamics, as well as the role of the immune system.

Features of CRC and CAC urge constant improvements in therapeutic approaches to these diseases. One of the most interesting and promising directions in the research on CRC treatment is the role and targeting of G-protein coupled receptors (GPCR) and their regulators (RGS). Within RGS proteins, canonical RGS and atypical RGS like AXIN can be distinguished. Within the abundant group of GPCR-targeting medications, cannabinoids have been constantly gaining interest as potentially anti-inflammatory and anti-cancer agents. Since they are already known as potent analgesics and anti-emetic and orexigenic drugs, their properties could be beneficial for patients in advanced stages of CAC, acting both as life-prolonging and palliative treatment. This review summarizes the current state of knowledge about the influence of RGS proteins on CRC carcinogenesis, including their potential usefulness for CAC treatment and diagnosis. 

## 2. Genetic Mutation Patterns in Sporadic CRC and CAC

Mutations altering genetic information are key processes determining cancer development. They can be classified as both somatic, acquired sporadically in the lifetime or inherited, leading to CRC development predisposition. Since it is estimated that inherited syndromes are responsible for about 5% of CRC cases [3], the majority of CRC patients (about 95%) suffer from CRC developed from somatic mutations.

Interestingly, one of the most important determinants of mutation patterns within CRC cells is the primary localization of the tumor. It was reported that CRC located in the cecum, ascending colon and two-thirds of the transverse colon (so-called right-sided tumors) have a worse prognosis and is connected with CpG island methylator phenotype (CIMP), microsatellite instability, more frequent mutations of B type rapidly accelerated fibrosarcoma (BRAF) and Ki-ras2 Kirsten rat sarcoma viral oncogene homolog (KRAS) genes. Left-sided tumors located in the fragment from the final third part of the transverse colon to the rectum generally have a better prognosis and are connected with more frequent displays of chromosomal instability [6].

The most important signaling pathway involved in CRC carcinogenesis, responsible for about 90% of tumors, is the wingless/integrated (Wnt)/adenomatous polyposis coli (APC)/β-catenin pathway. Physiologically, it is involved mainly in maintaining stem cells located in intestinal crypts [7]. The signaling route consists of Wnt glycoprotein, which interacts with FRIZZLED and lipoprotein receptor-related protein (LRP) receptor complexes, which results in the degradation of the β-catenin destruction complex that includes APC and AXIN proteins. This allows intact β-catenin to undergo translocation to the nucleus, where it acts as a transcription factor leading to the expression of genes responsible for cell proliferation. In fact, it was reported that in 92% of sporadic CRC, at least one regulator of Wnt signaling is altered, among which APC is the best recognized one; mutations decreasing APC activity led to an increase in the nuclear level of β-catenin since APC is involved in the destruction complex [8].

CAC is a specific subtype of CRC with characteristic etiology; CAC originates from chronic intestinal inflammation observed in the course of IBD [4]. In the course of these diseases, the pro-inflammatory cytokines and chemokines (e.g., IL-6) lead to the recruitment of immune cells that secrete oxidative enzymes, like myeloperoxidase (MPO) and the generation of oxygen and nitrogen reactive species (ROS and RNS), which induce mutations in epithelial cells. This leads to alterations in the functioning of intestinal epithelial cells cycle and affects their other properties, including migration, eventually leading to carcinogenesis and metastases [9]. It was found that mutation patterns observed in CAC differ from those in sporadic CRC [10]. Importantly, the examination of exome sequences in samples of CAC revealed a more abundant pattern of mutations in cell communication pathways and cell adhesion. One of the most significant differences is the earlier presence of p53 and delayed, less-frequent APC and KRAS mutations in CAC in comparison to sporadic CRC (13% vs. 20%) [10]. However, despite theoretically more spared APC function in CAC compared to sporadic CRC, the nuclear translocation of β-catenin is still significantly higher than in healthy enterocytes. The described findings emphasize the heterogenicity among CRC tumors that require the development of targeted treatment and diagnostic methods.

## 3. Role of Inflammation and Immune System in CRC Oncogenesis

Inflammation is the key driving factor in CRC formation; however, playing different roles depending on the CRC subtype; while CAC is originally driven by inflammation, in sporadic CRC, the systemic inflammatory response appears rather as a consequence than a cause of cancer development. More precisely, the association of inflammation and stages of CRC pathogenesis can be considered in three different groups [11]: (1) inflammation preceding carcinogenesis leading to CAC, (2) inflammatory response induced in the course of carcinogenesis and (3) later inflammatory activity resulting from chemo- and radiotherapy.

### 3.1. Inflammation Preceding Carcinogenesis

According to the statistical data, most significant environmental factors related to increased risk of CRC development include: family history (R = 1.79, 95% CI: 1.60–2.02), IBD (RR = 2.93, 95% CI: 1.79–4.81), diet (red meat consumption; RR = 1.13, 95% CI: 1.08–1.12) and obesity (RR = 1.10 per 8 kg/m^2^ increase, 95% CI: 1.08–1.12) [12]. All of these main factors act through chronic inflammation, which is a form of response to environmental stimuli. However, it was estimated that in the case of CRC, only up to 5% of cases originate from externally induced chronic inflammation. Another example of chronic inflammation is the reaction observed in IBD (Table 1). While the precise etiology underlying the inflammation in these diseases is still not fully understood, the most influential factors connected with IBD pathophysiology are genetic factors, intestinal microbiome alterations and disrupted intestinal barrier function. In fact, the profile of inflammatory response differs depending on the IBD type; in UC, the major CAC-predisposing IBD, the inflammation is driven mainly through Th2 cells, and tissue infiltration includes higher rates of granulocytes, while in CD, the inflammation is connected with Th1/Th17 phenotype and inflamed tissues are infiltrated mainly by lymphocytes [13,14,15,16]. 

The main mechanism of promoting tumorigenesis by inflammation is the generation of highly reactive ROS and RNS. These molecules cause mutations and nucleic acid disruptions directly or indirectly by decreasing the efficiency of the intestinal barrier and, therefore, increasing intestinal exposure to mutagenic factors, including microbes, which may sustain the inflammation. 

The other, more subtle way of connecting inflammation with cancerogenesis leads through cytokines’ crosstalk, especially interleukin-6 (IL-6), which is dependent on the nuclear factor kappa-light-chain-enhancer of activated B cells (NF-κB). In the experiment conducted by Zeng and colleagues [17] on intestinal tissue samples from CRC patients, it was showed that IL-6 expression correlates positively with CRC staging expressed in TNM (tumor, nodes, metastases), depth of invasion and presence of nodular metastases. On the other hand, a negative correlation was found with the level of tissue differentiation. Mechanistically, IL-6 is associated with pro-inflammatory and proangiogenic properties acting through signal transducer and activator of transcription 3 (STAT3), leading to increased expression of vascular epidermal growth factor 2 (VEGFR2) and regulation of the expression of genes involved in cell cycle control and metalloproteinases (e.g., MMP-1). Noteworthy, all these factors favor cancer growth in different stages, starting from initiation to promotion and progression, which makes IL-6 one of the most important cytokines in the CRC course.

### 3.2. Tumor-Associated Inflammation

Inflammation can also be observed accompanying sporadic CRC. In 2012, using a mouse model, Grivennikov et al. [18] described the loss of intestinal barrier efficiency, which is dependent on increased Wnt signaling pathway (e.g., loss of APC function). This leads to microbial-driven inflammation with further expression of pro-inflammatory and pro-tumorogenic cytokines, including IL-17 and IL-23. IL-23 is responsible for the recruitment of Th17 lymphocytes, which release effector cytokines, including IL-17, that result in the activation of pro-oncogenic factors like STAT3, NF-κB and β-catenin signaling routes. Moreover, Th17 cells display proangiogenic and anti-apoptotic properties and recruit myeloid-derived tumor cells, creating an immunosuppressant environment. All these features further promote cancer growth [19]. 

Another mechanism in which sporadic CRC may cause inflammatory response is through hypoxia that occurs as the result of rapid tumor growth with insufficient nutritional supply. The molecular marker of hypoxia is hypoxia-inducible factor 1α (HIF-1α) [20], which was already connected with CRC resistance to therapy with 5-fluorouracil in phosphoinositide 3-kinase (PI3K)/protein B kinase (Akt) and β-catenin-dependent manner. Moreover, it was reported that blockage of HIF-1α may suppress tumor growth and angiogenesis and improve the radiation sensitivity of CRC.

### 3.3. Inflammation Induced by CRC Treatment

Besides surgical treatment, currently available CRC treatment strategies involve both radio- and chemotherapy, which may alter inflammatory responses, often balancing between pro-inflammatory and immunosuppressive properties.

Radiotherapy (RT) using highly energetic radiation causes cancer cell death and irradiation of tumor microenvironment cells (TME), resulting in the release of ROS/RNS, damage-associated molecular patterns (DAMPS) and cytokines activating innate immune response (driven by dendritic cells and macrophages). The systemic inflammatory response is triggered after the subsequent migration of activated cells to the lymphatic structures located in non-radiated areas. Moreover, in the tumor area, the RT upregulates the adhesion molecules (including ICAM-1 and VCAM-1), which are also expressed in blood vessels that predispose to immune cell infiltration of the tumor microenvironment. On the other hand, RT also leads to immunosuppressant effects, including increased activity of cytokines such as transforming growth factor-β (TGF-β), adenosine, VEGF, colony stimulating factor-1 (CSF-1) and chemokine (C-C motif) ligand 2 (CCL2) resulting in the gathering of regulatory lymphocytes T (Treg) and macrophages with M2 phenotype.

Administration of chemotherapy may cause inflammation either as an undesirable complication or as an intended outcome. In the first case, the inflammation results from cells’ death in a similar mechanism as in RT; however, intended inflammation is an outcome of the administration of immune ‘checkpoint blockers’, including PD-1 (programmed cell death protein, e.g., pembrolizumab) and CTLA4 (cytotoxic T cell antigen 4, e.g., ipilimumab) inhibitors which may be effective in case of metastatic CRC with deficient mismatch-repair mechanisms and microsatellite instability pattern (MMR-deficient/MSI^high^) [21]. This therapy leads to the accumulation of short-lived CD8+ cytolytic lymphocytes [22], which are strongly associated with IBD and CRC microenvironment. Through this mechanism, checkpoint blockers enhance the anti-tumor immune response. Moreover, an additional outcome of this intended inflammation is the generation of long-lasting memory lymphocytes mediating anti-tumor responses in the future [23]. 

## 4. G-Protein Coupled Receptors

GPCRs are an abundant family of membrane receptors with about 900 representatives that exert their downstream effects through signaling dependent on G-protein. The range of physiological functions of GPCR is far-reaching and encompasses crucial elements of oncogenesis and its prevention, such as regulation of cell cycle and survival, responses to various endogenous- (e.g., hormones and neuroendocrine peptides, e.g., enkephalins) and exogenous stimuli (e.g., medications), angiogenesis and forming metastases [24]. Moreover, several types of GPCRs occur in the GI tract, including cannabinoid receptors, and it is estimated that about 50–60% [25] of commercially available medications target GPCR as the foundation of their mechanisms of action. 

**Figure 1:**
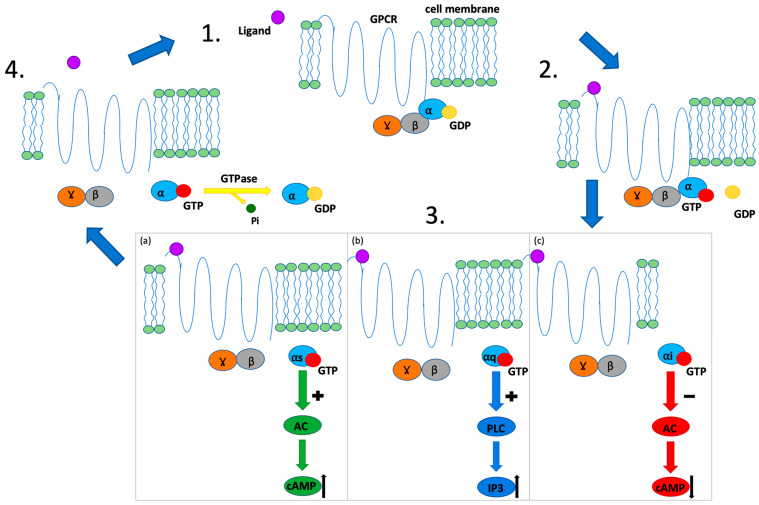
Scheme of GPCR function. 1. Resting state; ⍺, β and Ɣ subunits form G-protein. 2. Ligand binding: GTP replaces GDP binding to the ⍺ subunit. 3. Dissociation of the GTP-⍺ subunit complex: the complex interacts with its targets and causes different intracellular responses depending on the type of the ⍺-subunit: (**a**) G_⍺s_ subunit; stimulation of AC activity and increase in cAMP; (**b**) G_⍺q_ subunit; stimulation of PLC activity and increase in IP3; (**c**) G_⍺i_ subunit; suppression of AC activity and decrease in cAMP. 4. GTPase activity of RGS protein leads to GTP hydrolysis: the GDP-⍺ subunit complex is created again; ligand dissociates from the receptor; G-protein in the resting state will be formed. Abbreviations: GPCR; G-protein coupled receptor; GDP; guanosine diphosphate; GTP; guanosine triphosphate; α, β, γ; G protein subunits; Pi; phosphate; AC; adenylyl cyclase; cAMP; cyclic adenosine monophosphate; PLC; Phospholipase C; IP3; Inositol trisphosphate.

There are a few main types of G-proteins involved in GPCR signaling (Figure 1): Gα (including Gα_s_, Gα_i/o_, Gα_q/11_, and Gα_12/13_), Gβ and Gγ, which form the βγ dimer. The main mechanistic feature of Gα-proteins signaling is binding to guanosine diphosphate (GDP), which is switched to guanosine triphosphate (GTP) after receptor stimulation. Downstream effects may include (1) adenyl cyclase stimulation (Gα_s_) or inhibition (Gα_i_) and (2) the activation of phosphatidylinositol signal pathway, in which the Gα_q_ subunit recruits phospholipase Cβ (PLCβ), which produces secondary signaling particles: diacylglycerol (DAG) and inositol 1,4,5-trisphosphate (IP3) [26]. Because of the variety and omnipresence of GPCR, not surprisingly, they participate in inflammation in the course of the IBD and eventually CRC and, therefore, constitute potent pharmacological targets.

## 5. Selected GPCR in IBD and Their Connections to Carcinogenesis

In the course of IBD, the inflammation differs quantitatively (depth of inflammatory infiltration) and qualitatively (involved immune cell subpopulations), depending on the subtype of the disease. However, from a molecular perspective, similar GPCR-modulated signaling pathways are involved in this process.

One of the most promising groups of GPCR agonists are opioids, which exert their effector activities through opioid receptors (OR) µ, δ and κ (MOR, DOR and KOR, respectively) belonging to GPCR connected with G_i/o_ protein. Although within the GI tract they are typically associated with expression in muscles and neurons (submucosal and myenteric plexus), they are also present in immune cells, including lymphocytes and macrophages, being able to modulate their function [27]. 

It is generally believed that opioids exert mostly anti-inflammatory and suppressive effects on the immune system, causing a risk of severe bacterial infections accompanying prolonged opioid usage; however, available data are ambiguous. It is known that opioids modulate both types of immune responses: innate and acquired immunity. In immune cell lines (Jurkat, THP-1), the expression of MOP was inducible with exposure to pro-inflammatory cytokines (TNF-α, IL-1β and IFN-γ). Moreover, it was also suggested that morphine favors peripheral blood mononuclear cell (PBMC) differentiation towards Th2 type with increased production of Th2-related cytokines (IL-4, IL-5), which resembles the status of the immune system in the course of UC [28]. On the other hand, it was found that morphine causes insensitivity of the chemokines’ receptors (e.g., CCR1 and 2, CXCR1 and 2) on macrophages by their phosphorylation [29]. Moreover, morphine was reported to decrease macrophages’ ability to respiratory burst, increase phagocytosis and altogether increase intracellular bacterial growth in these cells. Morphine effects on lymphocytes include decreased mitogenic ability and quantity of lymphocytes B, suppressed activity of natural killers (NK) and lymphocytes T, as well as their shift towards Th2 phenotype [30].

KOR receptors were reported to be locally upregulated in intestinal inflammation, but most likely as a result of post-transcriptional or translational changes rather than de novo KOR synthesis [31]. Importantly, anti-inflammatory activity was also reported for KOR activation, including alleviation of in vivo colitis in mouse models confirmed by a decrease in colonic neutrophil infiltration pro-inflammatory cytokines levels, including IL-6 and TNF-α [32]. Moreover, selective KOR agonists such as U50,488H have well-documented anti-inflammatory properties, i.e., attenuation of edema and inflammatory pain. In in vitro on murine macrophages, it was shown that U50,488H dose-dependently decreased production of IL-12, with no impact on IL-10 release [33]. The anti-inflammatory properties of KOR agonists originate from various mechanisms, including the reduction of substance P (SP) and calcitonin gene-related peptide activity [34]. Although these observations were found in joint tissue in patients with arthritis, it is worth mentioning that the upregulation of SP and its receptors on immunoreactive fibers within the colon was already reported in UC as a pro-inflammatory factor responsible for the possible neurogenic mechanism of inflammation observed in UC [35,36]. Other anti-inflammatory mechanisms of KOR agonists include the inhibition of the Toll-like receptor 4 (TLR4)/NFκB signaling pathway, which was extensively studied in myocardial ischemia and reperfusion models [37].

Similar results were obtained for DOR agonists. In the experiment performed by Nagata et al. [38], DOR activation resulted in a decrease in pro-inflammatory markers IL-6 and nodular macrophages and an increase in Treg cells. 

Despite the fact that immunosuppression [39] is a risk factor for cancer development, MOR-related signaling was also independently linked with the activation of the pro-oncogenic signaling pathway involved in CRC formation; the PI3/Akt/mTOR (mammalian target of rapamycin) and Src (from ‘sarcoma’) pathways [39]. Notably, MOR antagonist and partial KOR agonist, nalmefene reduced cancer cell migration and downregulated pro-oncogenic signaling pathways, including calcium/calmodulin-dependent protein kinases II (CaMK II) and GSK-3β pathway. Moreover, it was suggested that CRC cells may produce endogenous DOR agonists to cause local immunosuppression [9,40].

Another group of biologically active compounds acting through GPCR is cannabinoids. They are a group of both endogenous fatty acid-derived compounds and synthetic medications that have already found an application in modern medicine as anti-vomiting drugs and orexigenic agents. Recently, more attention has been paid to the role of cannabinoids and their potential tumor-suppressive properties. Cannabinoids exert their physiological effects through interactions with cannabinoid receptors 1 (CB1) and 2 (CB2), which are GPCRs classified as Ga i/o-type and are thought to reduce cellular cAMP levels, ultimately leading to apoptosis and suppression of cancer development [41]. 

It was discovered that stimulation of cannabinoid receptors with their agonists (HU210 and JWH-133) attenuate inflammation in experimental models of colitis, including dextran sodium sulfate (DSS)-induced and dinitrobenzene (DNBS)-induced mouse models of colitis [42,43]. Moreover, the endogenous cannabinoid anandamide is increased in samples obtained from patients with UC compared to control [44], which is most likely caused by downregulated expression of cannabinoid degrading enzyme-free fatty acid amide hydrolase (FAAH) in the course of UC [45]. 

The majority of available data plead in favor of the anti-cancer properties of cannabinoids. Noteworthy expression of both types of CB receptors is increased in tumor samples compared to unchanged tissue [46]. However, the deletion of CB1 resulted in more rapid cancer growth in a genetic mouse model of CRC [47] in APC^Min/+^ mice, while treatment of Cnr^+/+^/APC^Min/+^ mice with CB1 agonist R-1 methanandamide resulted in tumor growth inhibition through downregulation of anti-apoptotic factor: survivin. These results suggest a suppressive role of cannabinoid signaling in oncogenesis and their upregulation within tumor tissue. Moreover, it was shown that in azoxymethane (AOM)-induced CRC, the application of cannabinoid agonist HU210 and inhibitor of endogenous cannabinoid-hydrolyzing enzyme (fatty acid amide hydrolase, FAAH) results in the decreased formation of precancerous aberrant crypt foci [48] and lesions in xenograft models [49]. Furthermore, cannabinoids were reported to block the ability of cell cycle progression in tumor cells, cause autophagy, activate apoptosis and suppress angiogenesis [50]. Noteworthy is that a well-established pharmacological tool, WIN 55,212-2, which is a CB1 and CB2 agonist, was also reported to have anti-cancer properties. In an experiment [51] on prostate cancer cells (PC3 and DU145), WIN 55,212-2 was reported to block the cell cycle in the G1 phase in a CB2-dependent manner through upregulation of p27 and downregulation of cyclin kinase 4 (Cdk4) that eventually led to the inactivation of pRb-dependent prooncogenic and cell-proliferative signaling. Unfortunately, WIN 55,212-2 was never assessed in the context of CAC. Summing up, all these findings suggest the beneficial activity of cannabinoids in CAC prevention and as a potential treatment.

Serotoninergic signaling also plays a crucial part in IBD pathophysiology. Serotonin (5-HT) is a monoamine acting as a neurotransmitter strongly connected to intestinal physiology. 5-HT interacts with the numerous 5-HT receptors, among which the majority belong to GPCR comprising members of all subtypes of canonical GPCR: Gα_i_, Gα_q/11_, and Gα_s_ [52]. The main source of 5-HT in the human organism is the 5-HT synthesis from tryptophan taking place in enterochromaffin cells located within intestinal mucosa; however, it is also produced by enteric serotonergic neurons located mainly in myenteric plexus [32]. 5-HT is strongly associated with various aspects of intestinal pathophysiology, including participation in intestinal microbiota regulation and pain sensation [53]. It was found that short-chain fatty acids, which are metabolites of commensal intestinal bacteria, increase the expression of tryptophan hydroxylase-1 (TPH1) that participates in 5-HT synthesis. In fact, in mice lacking TPH1 gene expression (*Tph1*^−/−^), the abundance of *Bacteroidetes* was higher; however, a low level of these bacteria was associated with higher IBD severity [54]. On the other hand, the orally administered mucosal-specific TPH inhibitors (LP-920540 and LX1032) were reported to attenuate trinitrobenzene sulfonic acid(TNBS)-induced mice model of colitis [55]. Moreover, 5-HT was shown to decrease the activity of β-defensin in both mouse colon and HT-29 cells, which may influence intestinal microbiota [56]. 

In the intestinal mucosa of IBD patients, 5-HT was reported to be increased compared to control as a result of IL-1β and LPS action on Toll-like/IL-1 receptor [57]. Interestingly, in the analysis performed by Manzella et al. [58], serotonin levels were more effective in the differentiation of CD activity stages than C-reactive protein concentrations. Moreover, increased 5-HT may partially explain manifestations of irritable bowel syndrome in the course of IBD [59]. 

Recently, 5-HT was directly connected with CRC cancerogenesis. It was shown that 5-HT, through interactions with its receptors 1B, 1D and 1F (HTR1B, HTR1D and HTR1F, respectively), directly supports the self-renewal of CRC stem cells in an acetylcholine-independent manner [60]. Moreover, the Authors found that the main source of 5-HT induced by tumor presence originates from enteric neurons expressing tryptophan-hydroxylase 2 (TPH2). In fact, mice lacking TPH2 expression developed significantly fewer tumors than mice with knocked-out TPH1. Interestingly, the effect of 5-HT on CRC stem cells was independent of the HTR1-associated Gα_i1_ subunit but relied on the interaction between HTR1 and AXIN1 impairing the AXIN1-APC association and formation of ß-catenin destruction complex. 

Altogether, GPCRs have a significant impact on IBD-related inflammation and its transformation towards CAC.

## 6. Regulators of G-Protein Signaling and AXIN

**Figure 2:**
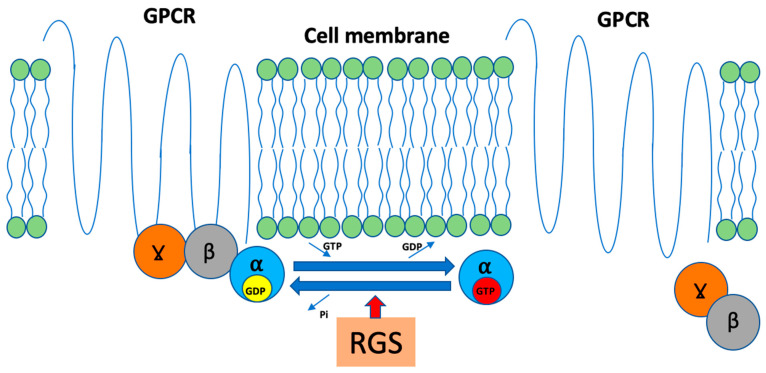
Scheme of RGS function. GPCR; G-protein coupled receptor, GDP; guanosine diphosphate, GTP; guanosine triphosphate, RGS; regulator of G-protein signaling, α, β, γ; G protein subunits.

Another group of proteins strongly involved in GPCR function is RGS. Typically, RGS proteins belong to GTPase-activating proteins (GAPs) that possess the ability to accelerate GTP to GDP hydrolysis (even up to 1000 times) and thus terminate G-protein signaling (Figure 2).

The key structural feature of RGS is the GAP domain (so-called RGS box), which is built from about 120 amino acids. This influences not only physiological GPCR function but also decreases the efficiency of drugs targeting GPCRs. However, the RGS proteins may also exert their activity in different mechanisms by forming scaffolds for receptors and their downstream associates, including G-proteins. Depending on the presence of additional functional domains in their structure, homology and Gα-specificity, the RGS proteins can be classified into five main families: R4, R7, R12, RZ and atypical RGS (Table 2) [61]. 

Several RGS proteins were already linked to CRC and are thought to influence oncogenic signaling pathways, resulting in alterations in their clinical implications. To date, several classical RGSs have been suggested to have clinical implications in CRC, including RGS2, RGS11, and RGS16.

Moreover, one of the most important RGS proteins in CRC pathogenesis is the atypical RGS, AXIN, which does not possess GTPase activity but instead is responsible for binding to APC protein [69]. 

## 7. RGS in IBD, CAC and Sporadic CRC Carcinogenesis

Even though the data directly binding the RGS proteins and CRC development are still limited, there are clues suggesting that connection. Since GPCRs are involved in key processes behind inflammation in IBD and possible further CAC development, the RGS as GPCR negative regulators are also expected to be substantially involved. Since the current state of knowledge on RGS proteins is less developed than in the case of GPCR, it is often insufficient to distinctly classify the impact of particular RGS proteins on the CAC course as positive or negative. The RGS proteins’ connections with types of GPCR are presented in Table 2. 

### 7.1. R4 Family: RGS1, RGS2, RGS4, RGS13 and RGS16

RGS1, RGS2 and RGS4 are classical RGS belonging to the R4 family and have one of the strongest connections to IBD among RGS proteins. 

RGS1 is strongly associated with intestinal inflammation mainly due to reports on its significantly elevated expression in lymphocytes (CD4+ and CD8+) accumulated in inflamed tissue in IBD patients. It was also found that RGS1 overexpression impairs chemotaxis towards C-X-C motif chemokine ligand 2 (CXCL2) in Jurkat cells (immortalized human lymphocytes T). In fact, the expression of RGS1 is significantly higher in lymphocytes T accumulated in tissues of IBD patients, suggesting that this alteration limits the scale of inflammation [32]. However, it is worth mentioning that RGS1 was also reported to be involved in the attenuation of MOR-related suppression of AC activity [70] and, therefore, may impact IBD, CAC and CRC formation in an opioid-dependent manner, especially through inhibition of inflammatory reactions. 

While RGS2 is commonly present in human tissues and acts on Gα_q/11_, the RGS4 interacting with Gα_i/o_ and Gα_q_ is expressed mainly in brain and cardiac tissues; however, it is also present in colonic smooth muscle cells [71,72]. Georgoussi and colleagues [73] created MOR and DOR receptors with modified carboxyl-termini and showed that RGS4 directly interacts with these receptors in a dose-dependent manner. Moreover, in the experiment with spinal RGS4, it was shown that its inhibition with intrathecal administration of CCG50014 attenuates inflammatory pain responses [74]. It is suggested that the anti-inflammatory effects of RGS4 inhibition may result from KOR-related signaling. It was shown that RGS4, together with RGS2, binds with activated KOR and suppresses KOR-related effects on cAMP and ERK1/2 [32].

However, RGS4 also affects cannabinoid signaling. Since Sutor et al. [75] performed the experiment directly linking RGS4 GAP activity with CB1 and CB2 receptors, it is assumed that inhibition of RGS4 could exert anti-inflammatory effects in mechanisms involving cannabinoid signaling. 

Lastly, RGS4 was also linked directly with the immune system. It was found that expression of RGS4 mRNA in colonic muscle cells is increased in pro-inflammatory conditions with the presence of IL-1β and was reduced by NF-κB inhibition [76].

There is proof linking RGS2 and RGS4 with cancerogenesis through protease-activated receptor 4 (PAR4). PAR4 is a GPCR whose activation requires proteolytic cleavage of the N-terminus. In CRC, PAR4 was reported to be upregulated, leading to increased phosphorylation of ERK1/2 and stimulation of epidermal growth factor receptor B-2. In fact, Zhang et al. [77] found that through the ERK1/2-dependent mechanism, PAR4 activation led to higher proliferation and migration of CRC LoVo cells. Moreover, the downstream effects of PAR4-related signaling include calcium mobilization and increased Rho activity. It was found that RGS2 and RGS4 inhibit signaling dependent on PAR4 in the presence of Gα_q_ and Gα_q_ or Gα_12/13_, respectively [71]. In addition, RGS2 and RGS4 interaction with PAR4 inhibits PAR4-related increase in expression of prooncogenic CRC-connected factors, including cyclooxygenase-2 (COX-2), activating transcription factor 3 (ATF3), basic transcription factor 3 (BTF3), SNAIL, zinc finger protein 91 (ZFP91) and *Leishmania* Heme Response 1 (LHR1) [71]. On the other hand, in another CRC cell line, the proliferation and migration of HT29 cells with knocked-out PAR4 expression were decreased. Concerning the clinical use of R4 RGS, Jiang et al. [78] found that downregulation of RGS2 mRNA expression is significantly correlated with poorer prognosis in CRC patients and may be involved in forming metastases that stay in line with the connection of RGS2 to the PAR4 signaling. Of note, RGS2, through suppressing KOR-mediated inhibition or ERK1/2 phosphorylation, may also act towards an increase in the anti-tumor activity of CD8+ cells. The lack of similar clinical findings for RGS4 may result from its expression restricted mainly to brain and cardiac tissues. 

Contrary to RGS2 and RGS4, another member of the R4 family, RGS16, does not interact with PAR4 directly; however, it is able to exert similar but weaker effects on it. Kim et al. [71] suggested that this phenomenon may result from the effect of RGS16 on Gα connected to the PAR4 without direct interaction with the receptor. On the other hand, recently, the effects of RGS16 on the anti-tumor activity of CD8+ cells were examined. Weisshaar et al. [79] demonstrated that RGS16 promotes anti-tumor CD8+ T lymphocyte exhaustion and limits their survival by interaction with scaffold protein IQ motif–containing GTPase-activating protein 1 (IQGAP1). The same group performed an analysis of a single-cell RNA dataset obtained from patients with metastatic melanoma [80] and found that higher RGS16 mRNA expression correlated with worse response to already mentioned immune therapy with PD-1 blockers and may be useful as a predictive marker of response to this treatment. In fact, the natural activity of RGS16 on immune cells exerts similar effects to the PD-1 blockage; however, contrary to these medications, it does not induce spontaneous autoimmune inflammation. Noteworthy, the clinical value of the RGS16 examination is already being considered. It was found that the RGS 16 [81] was upregulated in CRC and was suggested as a potential prognostic marker. Interestingly, RGS16 was also reported to be upregulated in PBMC and mucosa of UC patients, which correlated with UC endoscopic severity scales, including the Mayo scale and UC endoscopic index of severity (UCEIS) [63]. 

In summary, the impact of RGS16 on CRC development seems to be bidirectional since, on the one hand, it inhibits both prooncogenic signaling cascade depending on PAR4 and, on the other hand, limits the anti-tumor activity of the CD8+ lymphocytes; however, the impact of RGS16 on the immune system requires further studies.

Another R4 RGS, which is less described than others, is RGS13. In RNA-sequencing analyses performed by Verstockt et al. [62], RGS13 was identified as one of the genes potentially useful in predicting endoscopic remission of IBD in patients treated with vedolizumab. Interestingly, RGS13 was also reported to limit responses of B-type lymphocytes and the size of their germinal centers [82]. This finding suggests an anti-inflammatory role of RGS13, which is consistent with reports concerning vedolizumab treatment.

### 7.2. R7 Family: RGS6, RGS7, RGS9-2 and RGS11

Members of the R7 family, RGS7 and 9-2, were reported to regulate the sensitivity of MOR and DOR receptors in the nervous system by suppressing MOR endocytosis and MOR-related effects on cAMP production and ERK1/2 [32]. In the context of CRC, these RGS may have a similar impact on anti-tumor CD8+ lymphocytes and CRC as RGS2; however, there is no available literature linking RGS7 and RGS9-2 directly to any form of CRC. In fact, RGS7 was reported as one of the deleted genes in families with multiple cutaneous and uterine leiomyomatosis carrying a heterozygous deletion of 1q42-43. This mutation has been previously suggested as a cause of CRC susceptibility [64]. Notably, despite the deletions, none of the family members developed colorectal tumors.

However, in the case of RGS6, there are available proofs of its altered expression in different types of malignancies, including CRC and other cancers originating from ovaries, pancreas and breast. Luo et al. [83] found in human CRC tissue samples that RGS6 was downregulated in CRC tissues on both mRNA and protein levels compared to adjacent normal tissues. Moreover, the Authors managed to link decreased RGS6 expression with clinical parameters, including increased carcino-embryonic antigen (CEA) serum levels, enhanced TNM staging and its components independently: tumor size (T), forming nodular (N) and distant metastases (M), which suggests the anti-tumor properties of RGS6 activity. On the molecular level, RGS6 was reported [84] to antagonize the pro-oncogenic function of RAS protooncogene by promoting transcriptional coregulator (Tip60)-mediated degradation of DNA methyltransferase 1 (Dnmt1). Interestingly, RGS6 served as a scaffold protein using its RGS domain to associate directly with Tip60 and enable its interaction with Dnmt1. Even though these results were not obtained directly in the CRC context, RAS mutations are strongly connected to the clinical aspects of CRC management. Examination of RAS mutations is necessary for CRC patients in which administration of anti-epidermal growth factor therapy is considered since the efficiency of this treatment is strongly dependent on the presence of wild-type RAS [85]. Additionally, RGS6 can be linked with IBD and CAC through its involvement in the inhibition of detrimental 5-HT signaling in intestinal inflammation [32]. 

Contrary to the previously described RGS proteins, the RGS11 [86] is upregulated in CRC cells and was associated with resistance to oxaliplatin. Even though there are no available data linking RGS11 with CRC development, it is worth mentioning that RGS11 is already recognized as a lung cancer biomarker whose activity was correlated with the formation of metastases [87]. 

### 7.3. R12 Family—RGS10

The most significant representative of the R12 family is the RGS10, which displays anti-tumor activity. It was found that RGS10 participates in limiting the downstream effects of opioid agonist DAMGO [70] as well as 5-HT1A signaling [88] that links it with IBD. In fact, RGS10 was directly connected with IBD in an experiment concerning the overlap between Parkinson’s disease (PD) and IBD. In that experiment, reduced levels of RGS10 were detected in PBMC of PD patients. Moreover, the Authors showed that RGS10 deficiency in the RGS^−/−^ genotype aggravated colitis and disturbed the nigrostriatal dopaminergic system in mice [65]. Moreover, RGS10 was also directly linked with CRC. Calderon and Cacan [89] found that RGS10 is upregulated in a healthy colon in comparison to cancerous-altered colon tissues in which DNA methylation suppresses RGS10 expression. Finally, the Authors suggested that inhibition of DNA methylation could improve survival rates in CRC patients. 

### 7.4. RZ Family: RGS17, RGS19, RGS20

Representatives of the RZ family, RGS17, RGS19 and RGS20, were also found to substantially interact with opioid signaling by regulation of MOR sensitivity. It was reported that RGS17 and RGS20 participate in protein kinase C interaction with the C-terminus of MOR using their Z2 and Z1 domains [90]. There are limited data on RZ RGS in IBD. Recently, Suzuki et al. [66] identified RGS17 as the only significant gene related to mesalazine-induced fever and diarrhea in Japanese patients. On the other hand, RGS20 can be indirectly linked with IBD through clinically observable activity on MOR; RGS20 significantly decreased analgesic effects exerted by morphine [32], which suggests their impact on inflammation-related effects of MOR signaling. Furthermore, there are reports suggesting the impact of RGS17 on CB2 receptors; however, there are no data concerning intestinal tissues [67].

Unfortunately, there are no available data directly linking RGS19 and RGS20 with inflammation. However, there is some evidence connecting RZ RGS with cancers. Wang et al. [91] described that RGS19, but not RGS20, prevented Ras-induced oncogenesis in NIH3T3 cells and RGS19 knockout in H1299 non-small cell lung carcinoma cell line enhanced tumorigenesis. Moreover, RGS19 is one of the most significant interaction partners of GIPC1, which is involved in the trafficking of transmembrane proteins and regulation of cellular processes involved in oncogenesis, including proliferation, planar cell polarity, cytokinesis and migration [92]. Importantly, RGS19 was identified as a gene related to a higher risk of worse survival in CRC patients [93].

RGS20 was also connected with tumors. Yang et al. [94] performed an experiment on cancer cell lines HeLa, MDA-MB-231, H1299 and A549, which showed strong pro-oncogenic activity of RGS20 expressed as promoting cancer cell adhesion, aggregation and migration. Moreover, RGS20 expression induced metastatic profile of changes, including upregulation of vimentin together with downregulation of E-cadherin. 

Unfortunately, there are no available data on RGS17 in CRC; however, there are links with other malignancies, including ovarian [95], breast [96], lung [97], prostate [98] and nasopharyngeal cancers [99]. 

Even though there is still a lack of data on the direct assessment of RGS17, RGS19 and RGS20 in IBD and sporadic CRC based on their impact on opioid activity and involvement in processes in other types of cancers, it seems vital to cover this field in the future studies.

### 7.5. Atypical RGS—AXIN

Since AXIN’s RGS domain is responsible for binding APC protein, the main role of AXIN, which links it to CRC, is its involvement in β-catenin destruction complex within which it cooperates with already mentioned APC, glycogen synthase kinase3 (GSK3), casein kinase1 (CK1), and the E3 ubiquitin ligase component TrCP1 [24]. Through this complex, AXIN antagonizes the canonical Wnt signaling pathway, which stabilizes β-catenin. In fact, there are two AXIN proteins, AXIN1 and AXIN2, which are considered as functionally equal; however, with different expression patterns within tissues—while AXIN1 is ubiquitously expressed, AXIN2 is much more connected with developmental tissues. Moreover, AXIN2 is involved in a positive regulation loop with Wnt activators whose activity results in increased AXIN2 transcription upon nuclear β-catenin presence [100]. In fact, the involvement in the β-catenin destruction complex makes AXIN arguably the most important RGS in CRC in terms of its functionality.

The already-mentioned differences in mutation patterns between CRC subtypes suggest that the role of AXIN is more emphasized in sporadic CRC than CAC, at least at the earliest stages of the disease. On the other hand, it was already reported that the nuclear translocation of β-catenin is elevated in CAC. This is most likely a consequence of NF-κB-dependent activation of the canonical Wnt/β-catenin pathway through inhibition of GSK-3β. Moreover, similar effects on the Wnt/β-catenin pathway were observed in the context of ROS generation and their further impact on cellular signaling, especially activation of the PI3K/Akt pathway [68]. These results justify the theory about the equally strong involvement of AXIN in CAC prevention and treatment as in sporadic CRC. Unfortunately, there are no studies assessing AXIN and AXIN-targeting drugs in CAC. Recently, small-molecular AXIN stabilizers have been synthesized (e.g., KYA1797K), whose anti-cancer properties were demonstrated [101] in transgenic mice (C57BL/6J-Apc^Min/+^, B6.129S-Kras^tm3Tyj^, C57BL/6J-Apc^1638N^) as well as xenograft model. Since a similar study was not performed in the context of CAC, the pharmacological potential of AXIN-targeting drugs in CAC and its difference compared to other CRC subtypes remains uncertain. Therefore, we emphasize the need to fill this existing gap in knowledge. 

## 8. Future Perspectives

Although the state of knowledge about IBD, CAC and CRC is being constantly improved, the specifics of IBD-associated carcinogenesis still remain unknown. Recent progress in this field has been made by Wei Li and colleagues [102], who focused on the oncogenic human mucin 1 C-terminal (MUC1-C) protein. Its function is to activate wound healing processes such as proliferation and remodeling, which, if prolonged, may lead to cancer progression [103]. They conducted an experiment on transgenic CAC mouse models in which they were searching for the potential role of MUC1-C in linking inflammation and cancer. They found that MUC1-C was upregulated in the progression of colitis to dysplasia and carcinoma. Moreover, they showed that targeting MUC1-C suppresses the population of intestinal stem cells (ISCs) marked by leucine-rich repeat-containing G protein-coupled receptor 5 (LGR5 in human/Lgr5 in mice). Lgr5+ ISC’s function is to respond to damage [104] (e.g., in the course of inflammation) by regenerating cells, but they might also initiate colon cancer, which was demonstrated in mouse models [105,106]. Noteworthily, the experiment showed that targeting MUC1-C silences MYC, which also results in suppression of LGR5. Although further investigation is needed, these promising results make us view MUC1 as a reasonable target for future research.

Moreover, RGS is likely to be a promising pharmacological target. In the future, this kind of drug could be applied in CAC and CRC treatment, as well as in cancers affecting other organs. Studies showed that RGS might act as both suppressors and initiators depending on the RGS protein and the context of cancer, e.g., RGS2 and RGS10 inhibit the progression of ovarian cancer [107], while RGS19 promotes its growth [108]. Unfortunately, besides the already mentioned AXIN-targeting KYA1797K, there is little to no research regarding RGS-targeted cancer therapy. Joshua J. Steffan and colleagues conducted an experiment that showed the potential suppressive properties of Rab7 (a small GTPase regulating the transportation of lysosomes), which inhibits prostate cancer growth and invasion [109]. These results suggest that similar effects might be possible in trials regarding CAC and CRC. Therefore, we emphasize the need to focus on RGS-targeted therapy research. 

## 9. Conclusions

Despite constantly ongoing research on CRC, there is still a lack of safe and efficient therapeutic options. The heterogenicity of cancers and the overwhelming amount of intracellular signaling pathways involved in their pathogenesis may be considered both as a cause of further difficulties in optimizing the therapy and as a perspective to create new treatment strategies based on interactions within intracellular signaling. This strategy could lead to targeted therapy for patients with different needs depending on the stage of the disease. One of the most promising targets in research on CRC therapy is RGS proteins, whose role in CRC pathogenesis is gradually becoming more apparent. Importantly, the typical activity of RGS that terminates GPCR responses often results in the weakening of xenobiotics effects that may affect the efficiency of classical pharmacological treatment. This feature suggests a positive impact of RGS protein blockage. However, the atypical RGS, AXIN, does not possess such activity; instead, it uses its RGS domain to stabilize the β-catenin destruction complex. Since AXIN naturally prevents tumor growth and antagonizes inflammation-related activation of Wnt/β-catenin signaling, stabilization of AXIN seems beneficial in cancer therapy. Based on AXIN function and ability to antagonize β-catenin rather than exert direct cytotoxicity, we hypothesize that stabilization of AXIN could result in suppression of tumor growth without interference with the anti-cancer effect of other medications. Since the increase in β-catenin occurs in pre-cancerous lesions and at the very beginning of CRC pathogenesis, AXIN stabilization could be effective as a standalone therapy for patients in the early stages of the disease. Moreover, on the basis of the current knowledge, we suggest that combined therapy, including AXIN stabilization and opioid or cannabinoid activity, would be beneficial for patients in the advanced stages of the disease. Since opioids and cannabinoids have already known properties, including analgesic and additional anti-emetic orexigenic activity, a combination of those compounds with AXIN stabilizers would lead to the development of a survival-prolonging treatment strategy simultaneously fulfilling patients’ need for palliative aspects of the therapy.

## Figures and Tables

**Table 1:** Comparison of key features of inflammation in IBD.

Feature	UC	CD	Reference
Anatomical location	Large intestine, from left-sided colitis to pancolitis, with the possibility of backwash ileitis	Any part of the GI tract may be involved with ‘skip areas’ between inflammatory sites	[14,15,16]
Depth	Limited to mucosa and submucosa	The whole width of the GI tract wall can be affected by fistulae formation	
Immune cells phenotype and key cytokines	Th2 IL-4, IL-5, IL-10, IL-13	Th1/Th17 IL-2, TNF, IFN-γ, IL-17, IL-23	
CAC relative risk ratio	RR = 2.75 (95% CI: 1.91–3.97) Well-studied, generally considered higher than in CD	RR = 2.5 (95% CI: 1.3–4.7) Less studied, generally considered lower than in UC	[5]

Abbreviations: CAC; colitis-associated colorectal cancer; CD; Crohn’s disease; RR; relative risk; UC; ulcerative colitis.

**Table 2:** RGS families with their key features regarding connection with IBD and IBD-related GPCR in the context of possible CRC formation.

RGS Family	Representatives	Connection with IBD and IBD-Related GPCR	References
R4	RGS: 1, 2, 3, 4, 5, 8, 13, 16, 18, 21	OR: RGS1, RGS2, RGS4 CB: RGS4 5-HTR: RGS4 Other links with IBD: RGS13, RGS16	[32,62,63] [32,64] [32,65]
R7	RGS: 6, 7, 9–2, 11	OR: RGS6, RGS7, RGS11 CB: RGS7 5-HTR: RGS6 Other links with CRC: RGS7	
R12	RGS: 10, 12, 14	OR: RGS10 5-HTR: RGS10, RGS12 Other links with IBD: RGS10	
RZ	RGS: 17, 19, 20	OR: RGS17, RGS19 RGS20 CB: RGS17 Other links with IBD: RGS17	[32,66,67]
Atypical RGS	AXIN1, AXIN2	Prevents pro-inflammatory effects of Wnt/β-catenin pathway	[68]
